# Bimodal ECG and PCG Cardiovascular Disease Detection: Exploring the Potential and Modality Contribution

**DOI:** 10.1007/s10916-025-02245-5

**Published:** 2025-09-12

**Authors:** Alessia Calzoni, Mattia Savardi, Marco Silvestri, Sergio Benini, Alberto Signoroni

**Affiliations:** 1 University of Brescia, Department of Information Engineering, Via Branze 38, Brescia, 25123 Italy; 2 Isinnova srl, Via Berlinguer 2, Brescia, 25124 Italy; 3 Department of Medical and Surgical Specialties, Radiological Sciences, and Public Health, University of Brescia, Viale Europa 11, Brescia, 25121 Lombardia Italy

**Keywords:** Cardiovascular diseases, Electrocardiogram, Phonocardiogram, Transfer learning, Explainability, Bimodal learning, Multimodal fusion

## Abstract

Early detection of cardiovascular diseases (CVDs) is crucial for improving patient outcomes and alleviating healthcare burdens. Electrocardiograms (ECGs) and phonocardiograms (PCGs) offer low-cost, non-invasive, and easily integrable solutions for preventive care settings. In this work, we propose a novel bimodal deep learning model that combines ECG and PCG signals to enhance the early detection of CVDs. To address the challenge of limited bimodal data, we fine-tuned a Convolutional Neural Network (CNN) pre-trained on large-scale audio recordings, leveraging all publicly available unimodal PCG datasets. This PCG branch was then integrated with a 1D-CNN ECG branch via late fusion. Evaluated on an augmented version of MITHSDB, currently the only publicly available bimodal dataset, our approach achieved an AUROC of 96.4%, significantly outperforming ECG-only and PCG-only models by approximately 3%pts and 11%pts, respectively. To interpret the model’s decisions, we applied three explainability techniques, quantifying the relative contributions of the electrical and acoustic features. Furthermore, by projecting the learned embeddings into two dimensions using UMAP, we revealed clear separation between normal and pathological samples. Our results conclusively demonstrate that combining ECG and PCG modalities yields substantial performance gains, with explainability and visualization providing critical insights into model behavior. These findings underscore the importance of multimodal approaches for CVDs diagnosis and prevention, and strongly motivate the collection of larger, more diverse bimodal datasets for future research.

## Introduction

Cardiovascular diseases (CVDs) remain the leading cause of morbidity and mortality globally, significantly reducing quality of life and imposing substantial burdens on healthcare systems worldwide [1]. Early diagnosis is critical to mitigate their adverse effects, as delayed identification invariably escalates risks [2]. Given the inherent complexity of cardiac physiology, CVDs encompass diverse pathologies, including arrhythmias, valvular heart diseases, and coronary artery disease. While gold-standard diagnostic tools–such as echocardiography, computed tomography, and angiography–offer high specificity, their widespread adoption for large-scale screening is constrained by cost, limited accessibility, and concerns regarding radiation exposure [3]. In contrast, electrocardiography (ECG) and cardiac auscultation serve as widely utilized, low-cost, and non-invasive first-line methods for the early detection of pathological cardiac conditions and for guiding subsequent specific diagnostic examinations [2]. An ECG directly records the heart’s electrical activity, whereas a phonocardiogram (PCG)–the digitalized audio signal obtained via an electronic stethoscope during heart auscultation–captures the sounds resulting from mechanical vibrations due to cardiac function and blood flow. Despite their distinct modalities, these two signals are intrinsically related, each providing complementary insights into cardiac function, and neither alone suffices for a comprehensive CVDs diagnosis [3, 4].

In this scenario, Artificial Intelligence methods offer promising approaches for developing automated diagnostic systems, thereby supporting the early detection of CVDs in preventive healthcare settings. To date, most research efforts have concentrated on single-modality strategies. These methods have primarily utilized either ECG signals [5–8] to detect conduction or structural disorders, or PCG signals [9–11] to investigate valvular conditions. More recent research [3, 12–22] has explored the integration of both modalities to enhance diagnostic performance by leveraging their complementary information.

Despite the demonstrated effectiveness of bimodal data in CVDs prediction, progress in this area remains constrained by the scarcity of large datasets combining both PCG and ECG recordings. While transfer learning has already been applied to address this limitation [20, 21], its full potential, particularly in PCG analysis, remains underexplored. In particular, publicly available unimodal PCG datasets have not been fully leveraged and, since PCG signals are essentially audio recordings, employing audio data as the source domain in a transfer learning setting can potentially enhance PCG interpretation. Therefore, in this work, we investigate whether applying transfer learning by fine-tuning a model, pre-trained on audio signals, with the utilization of a larger volume of unimodal PCG data, can effectively improve PCG signal comprehension and enhance the ability to detect CVDs by integrating information from ECGs. Furthermore, the precise contribution of each modality to the final prediction often remains ambiguous, as this aspect has not been explicitly investigated in previous studies. To bridge this gap and to better understand each modality’s contribution, we employed explainability techniques and feature space visualizations, thereby shedding light on how the network interprets and integrates information from both signals.

In summary, the main contributions of this work are as follows:

1. A comparative literature analysis, specifically exploring existing limitations within current bimodal approaches for CVDs prediction.
2. An investigation into the potential of an optimized transfer learning strategy from audio data, aimed at enhancing PCG-based abnormality detection by fully leveraging all publicly available unimodal PCG datasets.
3. The development of a novel bimodal classification model integrating both ECG and PCG signals, confirming the effectiveness of multimodal analysis compared to single-modality approaches for CVDs diagnosis.
4. The quantitative application of explainability techniques to determine the relative contribution of each modality (ECG and PCG) to the final classification decision.
5. An analysis of a 2D-projection of the features learned by the model, offering insights into its strengths and inherent limitations.

This study extends a preliminary version of our work presented in [23]. The key advancements in this expanded version include the comprehensive integration of explainability analysis and feature visualization (corresponding to contributions 4 and 5, respectively), alongside a more extensive comparative literature review and refined results (points 1 and 3). While certain technical details from the earlier version have been judiciously omitted where not essential for conceptual understanding, the additions presented herein contribute to enhancing the interpretability, robustness, and overall insight into our bimodal diagnostic framework.

## Materials and Methods

### Datasets

This study utilizes three major unimodal PCG datasets, as well as the Massachusetts Institute of Technology Heart Sounds Database (MITHSDB). To the best of our knowledge, MITHSDB is currently the sole publicly available bimodal dataset comprising recordings from both healthy and pathological subjects. As for the unimodal PCG datasets, the PhysioNet/CinC 2016 dataset represented the first significant effort to address the scarcity of large and open databases of heart sound recordings. It includes 3,153 PCGs from 764 individuals, exhibiting an imbalance between normal and pathological cases, and is publicly accessible on PhysioNet [4, 24]. Yaseen’s database consists of 1,000 noise-free recordings, equally distributed across five distinct cardiac conditions (including the normal case), and is publicly available on GitHub [25]. The CirCor DigiScope database, collected from a pediatric population in Brazil, features 3,163 recordings from 942 subjects, with an almost equal distribution between normal and abnormal cases. Heart sound signals were recorded from up to four auscultation points, and the dataset is available on PhysioNet [24, 26, 27].

The bimodal dataset leveraged in this work is an augmented version of the MITHSDB, originally generated by Li et al. [3]. The original MITHSDB, part of PhysioNet/CinC, comprises 409 PCG recordings, 405 of which are coupled with simultaneous single-lead ECG signals, obtained from 121 subjects. Among these bimodal signals, 117 belong to the healthy group, while the remaining 288 are labeled as pathological, having been collected from patients diagnosed with mitral valve prolapse, benign murmurs, aortic disease, or other miscellaneous pathological conditions [4]. The extended version was created by segmenting the raw signals with a fixed window of 8 seconds. A window stride of 8 seconds was applied for abnormal recordings and 3 seconds for normal recordings to achieve a balanced class distribution. In total, this augmented version contains 1,975 recordings, 1,009 from healthy subjects and 966 from pathological ones, each with a fixed duration of 8 seconds [3]. This dataset can be downloaded from Zenodo [28]. In our work, since this augmented version of the MITHSDB is already divided into two distinct subsets with an 80/20 split, we retained the 20% portion as the test set. The remaining 80% was further divided into training and validation sets using another 80/20 split, ensuring that each patient appears in only one set. This resulted in 1,243 ECG-PCG recordings in the training set, 337 in the validation set, and 395 in the test set, with class distribution maintained across all splits.

### Comparative Analysis of Bimodal Studies

The first study demonstrating the advantage of combining both ECG and PCG signals dates back to 2019 [12]. Subsequent works have employed a range of methodologies, including traditional feature extraction methods [13–15] and deep learning models [3, 16–18]. Some approaches integrated manually extracted features as neural network inputs [19], while others incorporated transfer learning strategies [20, 21]. More recently, Mathew et al. explored self-supervised methods to pre-train a foundational model using a large private bimodal dataset [22]. Table 1 summarizes the main characteristics of these related works on bimodal studies.

Several key aspects emerge from existing bimodal model research, highlighting both challenges and opportunities for improvement. Most studies [3, 15, 17, 20, 21] indicate that ECG-only models tend to outperform PCG-only models when leveraging the MITHSDB in a single-modality setting. This underscores the need for further efforts to enhance PCG interpretability to improve the performance of bimodal models. Additionally, as frequently outlined in the literature [3, 18, 20, 21], a primary limitation in bimodal model development is the scarcity of adequate datasets, a problem that transfer learning has been applied to address. Vieira [21] found that a fine-tuning setting failed to outperform a feature extraction strategy from an ImageNet-pretrained model, likely due to the sole reliance on PhysioNet/CinC 2016 for the heart sound branch. Furthermore, Koike et al. [29] demonstrated that models pre-trained on audio data, particularly PANNs [30] on AudioSet [31], yield superior representations compared to knowledge transfer from image data. Based on these findings, the experiments conducted in this study aim to explore a more effective transfer learning approach by fine-tuning the PANNs-CNN14 model, pre-trained on AudioSet, with additional unimodal PCG data.

Direct performance comparison among all existing literature methods is hindered by several factors. Firstly, five contributions (see Table 1) rely on private databases rather than MITHSDB, and the predicted diseases do not always align with MITHSDB labels, making direct comparisons with our approach challenging. Variability in acquisition devices also contributes to performance discrepancies. For instance, MITHSDB was collected using the Welch Allyn Meditron electronic stethoscope, whereas private datasets employed by Li et al. [12, 16] utilized a cardiovascular function detection device (CVFD-II, Huiyironggong Technology Co.), and Mathew et al. [22] used the Eko Duo digital stethoscope. Such differences in sensor type and audio quality can significantly affect signal characteristics, further complicating direct cross-study performance comparisons. Among the remaining studies, three [14, 15, 17] reported high accuracy but suffered from class imbalance issues and did not provide AUROC (Area Under the Receiver Operating Characteristic) values. Similarly, Morshed et al. [18] observed a performance drop when increasing the test set size from 10% to 30% of the data, suggesting poor generalization ability. Despite these complicating factors, meaningful comparisons will be presented between our solution and the reference methods.

**Table 1:** Summary of studies that combined ECG and PCG signals for classifying pathological heart conditions

Study	Outcome	Dataset	Numerosity	Approach	Performance
[12]	CAD/non-CAD	Private augmented dataset	3900 samples from 195 subjects	Manual feature extraction and deep learning	Acc: 95.6% AUROC: 95.1%
[13]	Normal/Abnormal	Subset of MITHSDB	100 samples	SVM	Acc: 92.5% AUROC: 95.1%
[14]	Normal/Abnormal	Subset of MITHSDB	342 samples	SVM	Acc: 93.1%
[15]	Normal/Abnormal	MITHSDB	405 samples from 121 subjects	SVM	Acc: 86.4%
[3]	Normal/Abnormal	Augmented MITHSDB	1975 samples from 121 subjects	CNNs and LSTM	Acc: 87.3% AUROC: 93.6%
[16]	CAD/non-CAD	Private augmented dataset	3900 samples from 195 subjects	1D/2D-CNNs	Acc: 96.5%
[17]	Normal/Abnormal	MITHSDB	405 samples from 121 subjects	BiLSTM-GoogLeNet	Acc: 96.1%
[18]	Normal/Abnormal	Augmented MITHSDB	Not specified	1D-CNNs	Acc: 95.1% AUROC: 99%
[19]	Five classes (normal + 4 pathologies)	Private dataset	335 samples	Signal decomposition and Neural Network	Acc: 96.1%
[20]	Normal/Abnormal	Augmented MITHSDB	3496 samples from 121 subjects	Transfer learning	Acc: 87.7% AUROC: 93.8%
[21]	Normal/Abnormal	Augmented MITHSDB	Not specified	Transfer learning (ImageNet)	Acc: 82.8% AUROC: 91.3%
[22]	Low EF detection	Private dataset	221184 samples	Transformer based architecture	AUROC: 84.5%

Acc = Accuracy, AUROC = Area Under the ROC Curve, BiLSTM = Bidirectional LSTM, CAD = Coronary Artery Disease, CNNs = Convolutional Neural Networks, LSTM = Long Short-Term Memory, SVM = Support Vector Machine, AF = Atrial Fibrillation, EF = Ejection Fraction

### Experimental Pipeline

#### Model Settings and Configurations

As a first step towards the proposed bimodal analysis system, one-dimensional Convolutional Neural Networks (1D-CNNs) were developed using either ECG or PCG signals to classify normal and abnormal heart conditions. These unimodal models used data from the augmented version of MITHSDB. 1D-CNNs were a strategic choice due to their high ability to capture local and hierarchical spatial patterns through their receptive fields, which is particularly beneficial since abnormal heart conditions, such as valvular diseases, often present with localized and specific waveform features. The network architectures were designed with decreasing kernel sizes and receptive fields covering at least a complete average heartbeat. These choices support effective multiscale feature extraction while ensuring analysis of a full cardiac cycle, which we hypothesized as necessary for pathology detection. Each unimodal network comprised multiple convolutional blocks, with each block containing a 1D convolutional layer, batch normalization, a ReLU activation function, and a pooling layer. Table 2 summarizes the configurations of these convolutional blocks and provides information on model complexity, expressed as the number of trainable parameters.

The subsequent step involved leveraging all unimodal PCG datasets to perform transfer learning, employing the PANNs-CNN14 as the base model pretrained on audio data, which served as the source domain. The parameters of its initial layers, responsible for spectrogram generation and log-mel transformation, were modified to handle the fixed-size 8-second signals. Meanwhile, weights obtained from training the PANNs-CNN14 model on AudioSet [30] were loaded into the remaining part of the network. The original final fully connected layer, designed for multiclass classification across 527 AudioSet classes, was replaced with a multilayer perceptron. The entire network was either fully fine-tuned or trained with its first convolutional blocks frozen (either 1, 2, or 3 out of 6 blocks), using all combinations of the unimodal datasets. Subsequently, the fine-tuned model was used to evaluate performance on the MITHSDB PCG signals. In this phase, the first 4 or 5 convolutional blocks of the base model were kept frozen, while the remaining layers were further trained.

Finally, the bimodal model was created using a late fusion strategy. ECG and PCG embeddings, extracted from the best unimodal models (excluding their fully connected heads), were concatenated and then processed by a multi-layer perceptron to produce the final classification. In this setup, we tested two distinct strategies: fully fine-tuning the entire bimodal network, and keeping one or both unimodal branches frozen for embedding extraction. We evaluated both the network incorporating the simple convolutional PCG-only model and the architecture based on transfer learning.

**Fig. 1:**
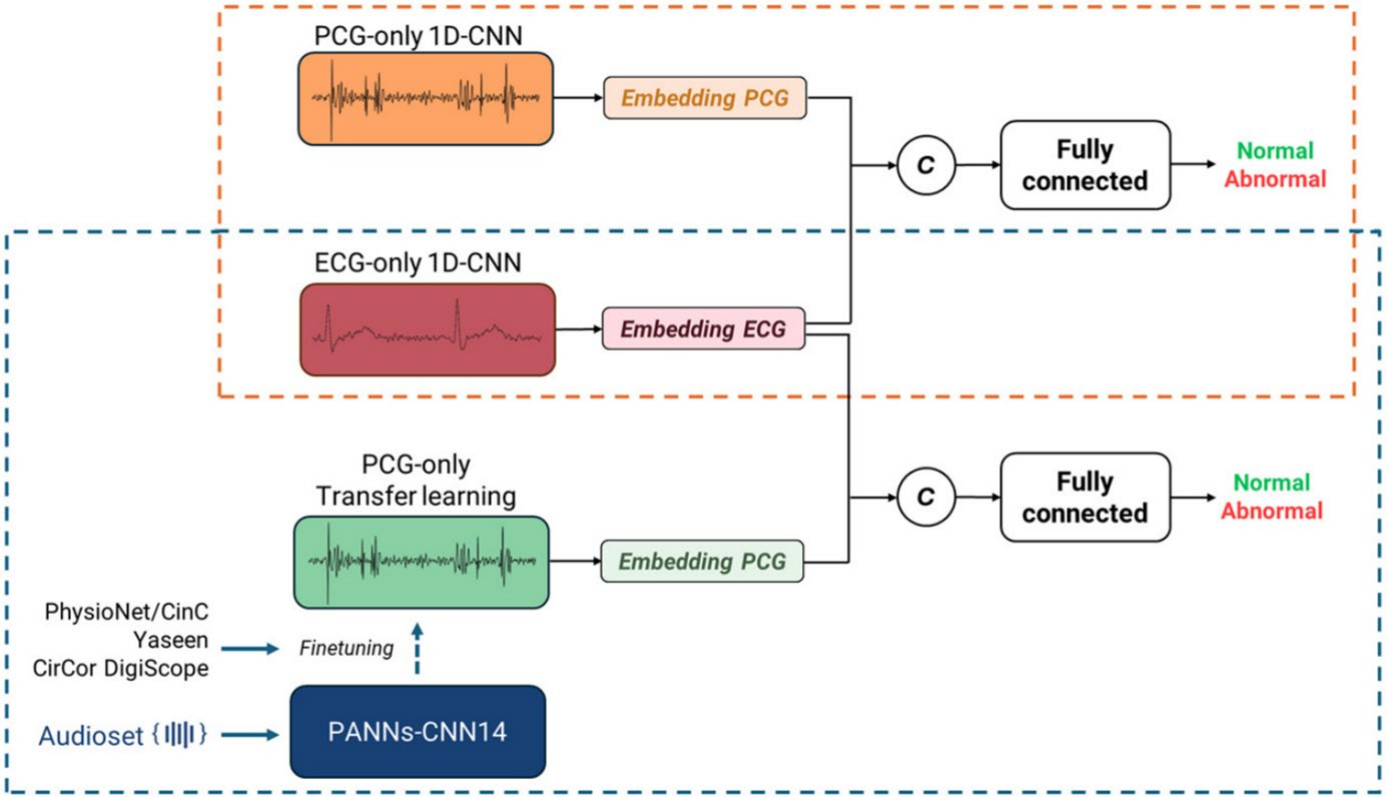
Overview of the proposed bimodal model combining ECG and PCG signals. The figure illustrates both strategies: with and without transfer learning. Embeddings from each modality are concatenated and used to generate the final prediction between normal and abnormal classes

Figure 1 provides an overview of the models, while further details concerning the architectures and preprocessing steps can be found in our preliminary work [23]. Given the limited size of the available bimodal dataset, we implemented some regularization strategies to mitigate potential overfitting risks. Dropout regularization was incorporated into the fully connected components of all architectures (with $P_{dropout}$=0.2) and treated as a hyperparameter for the 1D-CNNs configurations. Early stopping was applied during training based on validation accuracy monitoring, with a patience of 15 epochs to prevent continued training once performance plateaued. Finally, to enhance the robustness and reliability of our performance estimates, we employed bootstrap resampling with 100 iterations to generate 95% confidence intervals (CI) for all evaluation metrics.

**Table 2:** Different unimodal network configurations

# conv layers	# features maps	Kernel sizes	Pooling types	Dropout prob	# params
5	16, 32, 64, 128, 256	7, 5, 5, 3, 3	all avg/ all max/ both	0/0.2	153,923
6	16, 32, 64, 128, 256, 256	7, 7, k5, 5, 3, 3	all avg/ all max/ both	0/0.2	368,707
7	16, 32, 64, 64, 128, 256, 256	7, 7, 5, 5, 3, 3, 3	all avg/ all max/ both	0/0.2	372,995
8	16, 32, 64, 64, 128, 128, 256, 256	7, 7, 5, 5, 5, 3, 3, 3	all avg/ all max/ both	0/0.2	438,915

In the configuration with both pooling types, the first $\left\lfloor\frac{{\#}{\,{\rm conv~layers}}}{2}\right\rfloor+1$ pooling layers implement average pooling, while the remaining layers utilize max pooling

### Explainability and Visualization Analysis

To better understand how the model leverages information from each modality and to evaluate the internal representations learned by the bimodal network, we conducted a series of analyses focused on modality contribution and feature space structure. We employed three explainability methods: Grad-CAM (Gradient-weighted Class Activation Mapping), Multi-Layer Grad-CAM, and LIME (Local Interpretable Model-Agnostic Explanations), each adapted for time-series data. These techniques were selected to assess the relative contribution of each modality to the classification within the best bimodal model. First, we applied Grad-CAM to obtain activation maps from the final convolutional layer of each branch, weighting the feature maps by their corresponding gradient importance [32]. To address the inherent resolution loss in deeper layers, we incorporated Multi-Layer Grad-CAM [33]. This method enhances interpretability by aggregating activation maps from multiple layers. It generates layer-wise activation maps using absolute average gradient values and fuses them based on their correlation with the input sample. We adapted this approach by considering the last three convolutional layers and using the absolute Pearson correlation coefficient for weighting. Lastly, we adapted the LIME algorithm [34] for time series data. This involved segmenting the input and perturbing half of these segments by injecting noise sampled from their local distribution. A locally interpretable model was then trained to approximate the bimodal model’s behavior, assigning higher weights to perturbed samples more similar to the original input. These three methods were selected for their complementary strengths in quantifying modality contributions: Grad-CAM provides gradient-based importance measures, Multi-Layer Grad-CAM addresses potential information loss in deeper layers, and LIME offers model-agnostic local explanations that are robust to architectural choices, ensuring comprehensive assessment of modality relevance from multiple analytical perspectives. Since the signals were not temporally aligned, we couldn’t pinpoint specific influential time segments. Instead, we assessed modality importance using the mean of the normalized heatmap values as a global metric, providing a comparative measure of each modality’s contribution to the model’s decisions. Finally, to gain deeper insight into the best bimodal model’s capacity for discriminative and coherent feature representation, and to better understand its Limitations, we projected the test set embeddings into a 2D space using UMAP (Uniform Manifold Approximation and Projection) [35].

## Results

### Model Performance

**Fig. 2:**
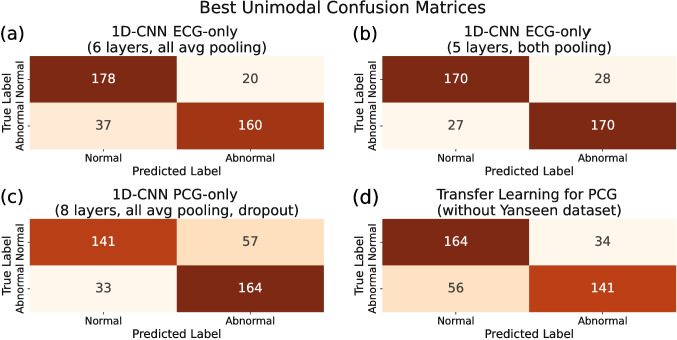
Confusion matrices of the best unimodal models on the test set of MITHSDB. (a) 1D-CNN ECG-only with the highest test AUROC; (b) 1D-CNN ECG-only with the highest test accuracy; (c) 1D-CNN PCG-only with the highest test AUROC and accuracy; (d) PCG-only transfer learning model with the highest test AUROC and accuracy

Figure 2 reports the confusion matrices for the best unimodal models. The optimal models were selected based on their AUROC and accuracy on the test set. For the 1D-CNN ECG-only model, the best-performing configuration comprised either six convolutional blocks with average pooling (Fig. 2a) or five blocks combining max and average pooling (Fig. 2b), with AUROC and accuracy serving as the respective selection criteria. Regarding the PCG-only model, both maximum AUROC and accuracy were achieved using eight convolutional blocks with only average pooling and dropout (Fig. 2c). Finally, in the transfer learning setting, the best model, which yielded both maximum accuracy and AUROC, resulted from a fine-tuning with all PCG recordings (excluding those from Yaseen’s dataset) and a subsequent training with MITHSDB data, with the first four convolutional blocks frozen (Fig. 2d). The exclusion of Yaseen’s dataset was motivated by experimental results showing that its inclusion during fine-tuning negatively impacted the downstream performance on MITHSDB data.

**Fig. 3:**
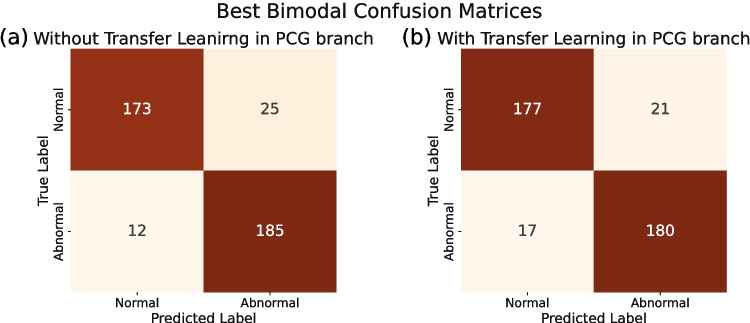
Confusion matrices of the best bimodal models on the MITHSDB test set: (a) with 1D-CNN in the PCG branch; (b) with transfer learning in the PCG branch

The confusion matrices for the best-performing bimodal models are shown in Fig. 3. These optimal bimodal configurations were selected based on both test accuracy and AUROC. Figure 3a corresponds to the setup using the 1D-CNN PCG-only model for the PCG branch, while Fig. 3b presents results when transfer learning was applied for PCG embeddings’ extraction. In both scenarios, the ECG branch employed a six 1D-convolutional blocks architecture, and embeddings were extracted with both branches frozen. Tables 3 and 4 summarize the corresponding performance metrics as mean values with 95% CI, obtained via bootstrap from the best-performing model selected based on the highlighted metric. Moreover, DeLong’s test was used to compare AUROC values between unimodal and bimodal models. All unimodal models showed significantly lower AUROC compared to the bimodal models (p<0.001), while no significant difference was found between the two bimodal configurations (p=0.32).

**Table 3:** Summary of the best unimodal model performances

Configuration	Acc	AUROC	Prec	Recall	Spec
1D-CNN ECG-only;5 layers, both pooling types	**86.1 %** **[83.8–88.1]**	92.9 %[90.7–95]	85.7 %[78.7–89.8]	86.8 %[83.1–91.2]	85.6 %[80.7–89.7]
1D-CNN ECG-only;6 layers, all avg pooling type	86 %[82.5–89.9]	**93.2 %** **[90.8–95.4]**	89.1 %[85.1–93.2]	81.9 %[76–88.4]	90 % [86.9–93.1]
1D-CNN PCG-only; 8 layers, both pooling types, dropout	**77.3 %** **[73.9–81.1]**	**82.8 %** **[78.3–86.7]**	74.6 % [70.2–79.3]	82.8 % [77.4–89.1]	71.7 % [67.7–76.9]
Transfer learning PCG	**76.9 %** **[74.2–80]**	**85.5 %** **[82.3–87.8]**	80.3 % [73.8–85]	70.7 % [65.5–75.1]	83 % [79.9–86.5]

For each model, mean values with 95% CI are reported, computed via bootstrap resampling with 100 iterations. The metric used to select the best-performing model is highlighted

**Table 4:** Summary of the best bimodal performances with or without implementing transfer learning into the PCG branch

ECG Model	PCG Model	Acc	AUROC	Prec	Recall	Spec
1D-CNN 6 layers; frozen	Without transfer learning; frozen	**90.3 %** **[87.3-92.9]**	**96.4 %** **[94.9-97.4]**	87.5 % [82.4-92]	93.8 % [90.2-97.8]	86.9 % [83.2-90.2]
1D-CNN 6 layers; frozen	Transfer learning; frozen	**90.5 %** **[87.3-93.2]**	**95.7 %** **[94.2-97.1]**	89.6 % [84.9-93.1]	91.5 % [88.8-94.5]	89.4 % [84.6-92.8]

For each model, mean values with 95% CI are reported, computed via bootstrap resampling with 100 iterations. For each model, the metric value used to select the best one is highlighted

### Explainability and Visualization

**Fig. 4:**
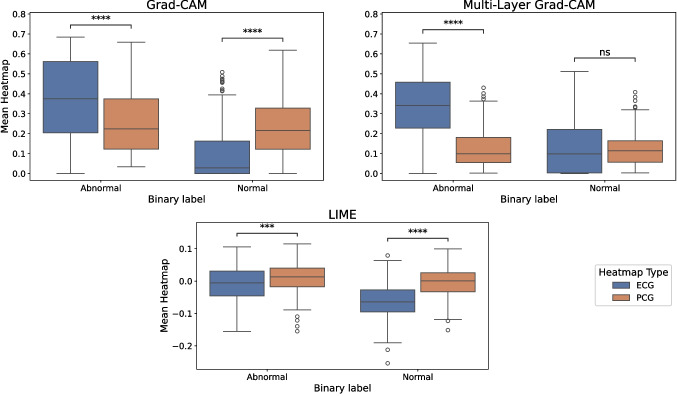
Comparison of the three explainability methods’ results. For the Wilcoxon signed-rank test: ns: 5e-02 < p $\le$ 1, *: 1e-02 < p $\le$ 5e-02, **: 1e-03 < p $\le$ 1e-02, ***: 1e-04 < p $\le$ 1e-03, ****: p $\le$ 1e-04

As mentioned in Section Explainability and Visualization Analysis, since the signals are not always temporally aligned, it is not possible to identify which specific time segments are most influential for the prediction. Instead, we utilized a global metric to assess the overall modality contribution and gain insights into the network’s decision-making process. The mean distributions of PCG and ECG heatmaps, normalized by the global maximum, are presented separately for each binary class. Figure 4 shows the boxplots corresponding to the different explainability techniques, along with statistically significant differences assessed using the Wilcoxon signed-rank test. For the normal class, all three methods consistently indicated that the median of the PCG heatmap means was higher than that of the ECG. Conversely, for the abnormal class, two out of three techniques revealed the opposite trend, with ECG heatmap medians exceeding those of the PCG. Clinically, the distinct modality shift observed offers a deeper understanding of the model’s diagnostic reasoning. Such insights can indicate which physiological signals are given greater importance, potentially guiding further and more targeted examinations.

**Fig. 5:**
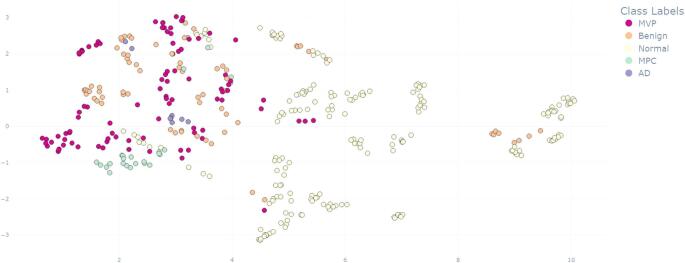
UMAP representation of the test set embeddings generated by the optimal bimodal model, showing the distribution of cardiac conditions in the learned feature space. MVP: mitral valve prolapse, Benign: benign murmurs, AD: aortic disease, MPC: miscellaneous pathological conditions

Figure 5 displays the 2D-projection of the test set embeddings obtained from the best-performing bimodal model using UMAP. The figure illustrates the distribution of different cardiac conditions in the embedding space. In the binary classification setup, the abnormal class aggregates several pathological subtypes, including mitral valve prolapse, benign murmurs, aortic disease, and other miscellaneous pathological conditions, as reported in Section Datasets. The visualization highlighted a clear separation between the normal class and pathological conditions, with only minor overlap, primarily involving benign cases, which accounted for most false negative predictions (10 out of 12). In contrast, the 2D projected representation did not reveal strong separability among the different abnormal conditions.

## Discussion

Our findings concerning the unimodal approach generally align with most prior works. Specifically, when using the MITHSDB in a single-modality setting, the best models developed with ECG signals consistently outperformed those trained solely on the corresponding PCG recordings. Despite our efforts to refine the transfer learning setting with several improvements, it did not enhance PCG interpretability as much as we expected compared to simpler 1D-CNNs models. This suggests that even a more refined transfer learning approach does not fully compensate for the limitations posed by the scarcity of bimodal data, underscoring the critical need for additional research and data collection in this area.

The observed improvements in test performance within the bimodal setting support the findings in Literature, highlighting that the complementary nature of ECG and PCG signals contributes to a more comprehensive understanding of cardiac conditions. Indeed, our bimodal approach achieved an overall AUROC of 96.4%, surpassing the performance of the ECG-only and PCG-only models by approximately 3 and 11 percentage points, respectively. As discussed in Section Comparative Analysis of Bimodal Studies, direct comparison with all existing methods is not always feasible. However, our best-performing bimodal configuration achieved a higher AUROC than other bimodal approaches operating on variants of the MITHSDB database, and it significantly surpassed [3]–the only study working on the exact same data–across all measured performance parameters, as detailed in Table 5.

Our analysis, conducted through different explainability methods, reveals a distinct modality shift: PCG features primarily influence normal-case predictions, while ECG features dominate in pathological ones. This behavior indicates how the network automatically prioritizes the signal most strongly associated with each classification. By dynamically balancing these inputs, our bimodal architecture captures a broader spectrum of cardiac patterns than either modality alone. Furthermore, visualization analysis shows that normal samples form a compact, well-separated cluster in the latent space. A minor overlap was observed with benign murmurs–conditions that may exhibit acoustic similarities to normal heart sounds. Crucially, these misclassifications are limited and acceptable, particularly within a screening context. Overall, this confirms the model’s reliability in confidently recognizing healthy individuals while preserving sensitivity to a wide range of cardiac abnormalities. While these analyses are currently constrained by the limited size and diversity of the available dataset, they offer valuable initial insights. We expect that access to larger and more varied datasets will enable deeper and more conclusive observations in future work.

Despite the promising results achieved by the proposed bimodal model, several limitations must be acknowledged. First, the model’s generalizability remains limited due to reliance on a single bimodal dataset with limited sample size. Specifically, our findings are based solely on the MITHSDB dataset and were not validated using external datasets. This constrains the broader applicability of our results and raises concerns about potential overfitting despite the regularization strategies employed. While these techniques, such as dropout, help in reducing variance and improving generalization estimates, they cannot fully compensate for the lack of data diversity. A more comprehensive data acquisition process is therefore crucial to assess the model’s adaptability in real-world scenarios, particularly across diverse patient populations. Moreover, as initially demonstrated by Mathew et al. [22], self-supervised learning architectures trained on large-scale datasets have shown strong potential to generalize across multiple diagnostic tasks. These findings underscore the importance of not only architectural advancements but also the availability of rich, multimodal datasets for developing scalable and reliable decision-support systems in real-world healthcare settings. Additionally, the feasibility of deploying such systems depends on the availability of synchronized acquisition tools and their standardized integration into routine clinical workflows. This also involves adapting existing hospital systems and diagnostic protocols, as simultaneous ECG and PCG acquisition is not yet common practice. In this context, real-world data acquisition presents several challenges, particularly in maintaining consistent signal quality and ensuring reproducible evaluations across different settings.

**Table 5:** Performance comparison on the test set with related works

Author	AUROC	Accuracy	Recall	Specificity
Chakir et al. [13]	*95.1%*	*92.5%*	*92.3%*	*92.9%*
Hettiarachchi et al. [20]	*93.8%*	*87.7%*	*87.7%*	*87.5%*
Vieira [21]	*91.3%*	*82.8%*	*93.1%*	*57.1%*
Li et al. [3]	93.6%	87.3%	90.3%	84.5%
Our model	**96.4%**	**90.6%**	**93.9%**	**87.4%**

Italicized entries refer to studies that are reasonably comparable in terms of using the MITHSDB dataset and focusing on binary classification, but do not use the exact same data splits as this study

## Conclusions

In this work, we conducted an in-depth analysis of prior research concerning the use of ECG and PCG signals in developing machine learning models for early CVDs detection. We explored a promising transfer learning approach, leveraging publicly available unimodal PCG datasets to address the challenge of insufficient bimodal ECG-PCG data. However, our findings indicated that this approach does not fully overcome the limitations posed by the scarcity of adequate bimodal datasets, highlighting the persistent need for further efforts in this area. Nevertheless, our results confirmed the effectiveness of a multimodal approach, demonstrating that combining the complementary insights from ECG and PCG signals can significantly enhance CVDs detection compared to single-modality approaches. Moreover, our explainability analysis explored the contribution of each modality and revealed that the model relies more on PCG information to recognize the normal condition and on ECG features to identify abnormalities. The UMAP visualization of embeddings further confirmed a distinct clustering of healthy versus pathological cases, enhancing interpretability for clinical deployment.

Overall, our work demonstrates that multimodal architectures can deliver superior performance in low-cost, non-invasive screening for CVDs. It also underscores the critical need for expanded and diverse bimodal datasets to fully realize their potential in large-scale CVDs prevention.

## Data Availability

All data used in this study are publicly available. The PhysioNet/CinC 2016 dataset is available at https://doi.org/10.1088/0967-3334/37/12/2181. The Yaseen’s database is available at https://doi.org/10.3390/app8122344. The CirCor DigiScope database is available at https://doi.org/10.13026/tshs-mw03. The augmented version of the MITHSDB is available at https://doi.org/10.5281/zenodo.4263528.

## Code Availability

The model architectures and weights are available from the corresponding author upon reasonable request.
